# Updating estimates of the number of UK stroke patients eligible for endovascular thrombectomy: incorporating recent evidence to facilitate service planning

**DOI:** 10.1177/23969873211059471

**Published:** 2021-11-16

**Authors:** Peter McMeekin, Darren Flynn, Martin James, Christopher I Price, Gary A Ford, Philip White

**Affiliations:** 1 Faculty of Health and Life Sciences, School of Health, Communityand Education Studies, 5995 Northumbria University, Newcastle upon Tyne, UK; 2 5462 Royal Devon & Exeter Hospital, Exeter and University of Exeter Medical School.; 3 Research Group, Newcastle University 5994 . Newcastle upon Tyne, UK; 4 Institute of Neuroscience (Stroke Research Group), 5994 Newcastle University, Newcastle upon Tyne, UK

**Keywords:** Thrombectomy, ischaemic stroke, advanced imaging, service planning, eligibility

## Abstract

**Introduction:**

Endovascular thrombectomy (EVT) is a highly effective treatment for acute ischaemic stroke due to large arterial occlusion (LAO). To support decisions about service provision, we previously estimated the annual UK population eligible for EVT as ∼10% of stroke admissions. Since then, several trials have produced evidence that could alter these figures. We update our estimates considering information from studies and trials reporting 2018–2021 on incidence, presentation time and stroke severity and consider the possible impact of predicted demographic changes in the next 10–20 years.

**Patients and Methods:**

We produce an updated decision tree describing the EVT eligible population for UK stroke admissions. One-way sensitivity analyses (using upper and lower confidence intervals for estimates at each branch of our decision tree) were used to identify where further research evidence is necessary to increase certainty around estimates for numbers of EVT eligible patients.

**Results:**

The updated estimate for the number of UK stroke patients eligible for EVT annually was between 10,020 (no advanced imaging in early presenting patients) and 9,580 (advanced imaging in all early presenting patients), which compared with our estimates in 2017 is a minimal reduction. One-way sensitivity analyses established that enhanced evidence about eligibility for milder strokes, ASPECTS scores and pre-stroke disability are offset by evidence regarding a lower incidence of LAO. Importantly, predicted increases in life expectancy by 2040 may increase thrombectomy need by 40%.

**Discussion:**

Information from additional randomised trials published during 2018–2020 with updated estimates of LAO prevalence had a minimal impact on overall estimates of stroke patients eligible for EVT in the UK. Ongoing research into the benefits of EVT for patients with mild stroke or European Stroke Journal For Peer Review lower ASPECTS scores has the potential to increase the estimates of the eligible population; future need for EVT will increase with the ageing population.

**Conclusion:**

Our updated analyses show overall numbers eligible little changed, but evidence from ongoing trials and demographic changes have the potential to increase the need for EVT significantly.

## Introduction

Clinical trials show that endovascular thrombectomy (EVT) is an effective treatment for acute ischaemic stroke causing large artery occlusion (LAO) with or without intravenous alteplase.^1–11^ In 2016, the Highly Effective Reperfusion Evaluated in Multiple Endovascular Stroke trials (HERMES) individual patient meta-analysis found that for every three patients treated with EVT, one would have reduced disability by at least one level on the modified Rankin Scale (mRS).^12^ EVT presents major challenges in many health care systems as it is typically carried out by neurointerventionists with anaesthetic and other specialist staff support. It requires substantial imaging infrastructure: rapidly performing computed tomography angiography (CTA), with or without advanced imaging by perfusion-computed tomography (CTP), magnetic resonance imaging techniques (MR) or multiphase CTA Collateral Scoring (mCTA-CS) in a centralised model of hyperacute stroke care. Therefore, significant planning and investment is needed in most health care systems to establish capacity to deliver EVT to those stroke patients most likely to benefit.

In 2017, we previously estimated the UK eligible population for EVT to estimate demand and inform service reconfiguration and estimate the annual demand for EVT in the UK, regardless of geographic or service constraints such as non-existent care pathways or a lack of imaging and EVT facilities.^13^ Subsequent studies and trials (DAWN, DEFUSE-3 and Manceau et al. ^9–11^) have added to the evidence base about patients who could benefit from EVT. Because of the number of ongoing trials^14–18^ into the effectiveness of EVT in other patient subgroups, many of which have been delayed by the COVID-19 pandemic, we re-examined our previous decision tree to produce an updated estimate of the proportion of all stroke patients eligible for EVT. We sought to quantify uncertainty around key nodes in the decision tree to enable planers to determine the effects of any new information on estimates of the eligible population on prevalence and effectiveness of EVT in sub-populations. Furthermore, we considered future stroke populations in terms of numbers and age profile in our sensitivity analyses looking 10–20 years ahead.

## Patients and methods

We reviewed estimates from our previous study based on national registry data from the prospective Sentinel Stroke National Audit Programme (SSNAP) for England, Wales and Northern Ireland^19^ and adjusted for Scotland using data from the Scottish Stroke Care Audit (SSCA)^20^ and produced an updated estimate of the number of UK patients hospitalised annually with acute stroke.^15^ The tree estimated eligibility by evidencing exclusions at progressive stages of the typical acute care pathway beginning with imaging to determine stroke type (ischaemic) followed by severity (National Institutes of Health Stroke Scale score; NIHSS) and time related eligibility for treatment. The tree then splits into early presenters and late presenters, with early presentation defined as within the IVT licence criteria. Eligibility in late presenters is determined using advanced imaging as well as clinical criteria. In addition to clinical and CT imaging criteria, we also allowed for the possibility of advanced imaging for early presenters for the purpose of excluding those without salvageable brain tissue. We reviewed evidence at each point in our previously published decision tree, updating its structure and numbers, as necessary, by expert consensus.

One-way sensitivity analyses (using plausible intervals for our estimates at each decision point (node) of the decision tree) were performed to identify branches of the tree that would benefit from additional evidence to increase certainty around the estimates for overall eligibility. The outputs from these analyses are presented graphically in a tornado diagram showing the impact of numbers eligible for EVT as a function of the upper and lower prediction intervals for each node of the tree. The decision tree was created in Excel and is provided as Supplementary material in Online Supplement File.

## Results

### Review of new evidence and change to nodal decision points

**Figure 1.**
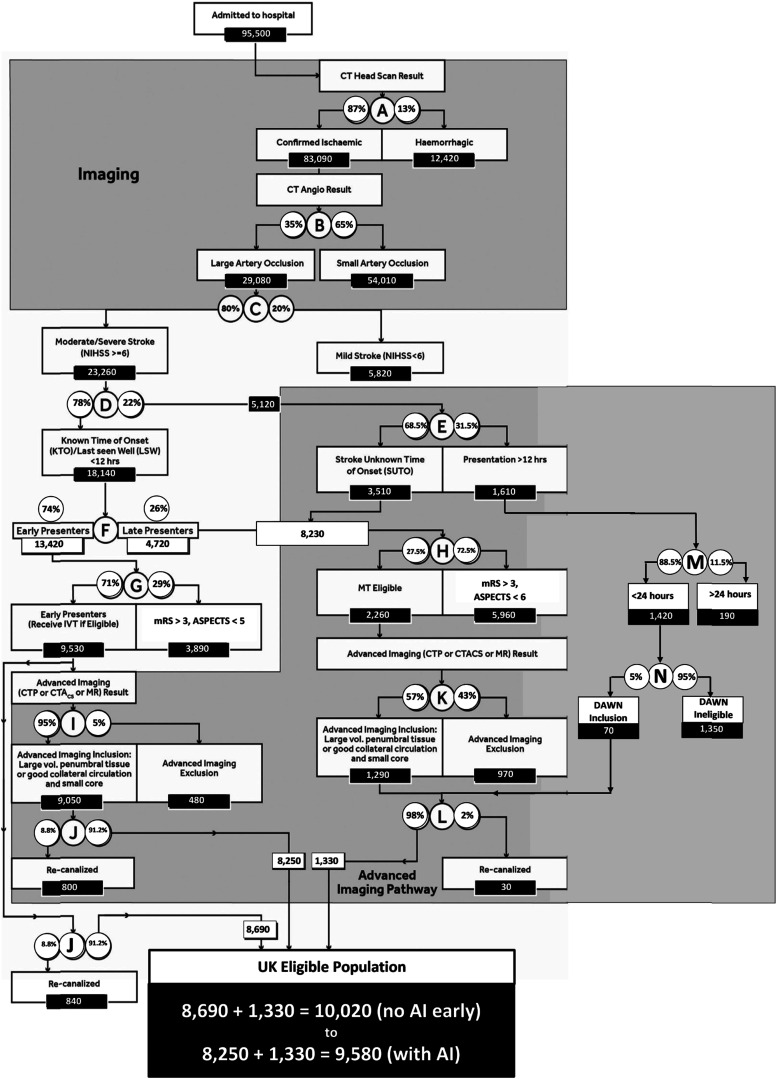
Eligible Population (a; Total UK population including those deemed to be geographically inaccessible. b: Confirmed infarcts, excluding ∼2% of patients whose status is unconfirmed. Besides cerebral infarcts, most acute subdural haematomas would also not be entered in to SSNAP nor SSCA. c: Includes basilar artery occlusions eligible for treatment if presenting within 12 h. Others are assumed eligible unless they meet any subsequent exclusion. d: ‘Early presenters’ (those presenting within licenced IVT window), late presenters or SUTO (4.5–12 h or last known well up to 12 h ago)). Note: Patients within the large lower grey shaded box are all dealt with by advanced imaging (8250 + 1330); those who are early presenters (8250 on the left-hand side) can bypass that step.

The updated decision tree is presented in Figure 1. In a 2019 review into the epidemiology, natural history and clinical presentation of large artery stroke published in 2019, Rennert and colleagues estimated that the hospital incidence ranged between 28% and 40%.^21^ Therefore, the mean incidence they reported, 35%, was used in our updated tree. This is lower than the previous estimate (40%) based on the earlier STOP-Stroke study^22^ and the more selective HERMES trial meta-analysis.^12^ The effect of this change was to lower the estimated eligible population, at node B from 33,240 to 29,080.

We previously estimated that (at node G), amongst early presenting (within the IVT licence window) moderate to severe strokes, 34% would be excluded from EVT because of clinical and/or imaging exclusions (originally a pre-stroke modified Rankin score (mRS) of 3 or more, or an Alberta Stroke Program Early CT Score (ASPECTS) of 5 or less). Proportions of patients with an ASPECTS 0–5 score was based on the Interventional Management of Stroke (IMS)-3 trial CTA positive subgroup.^23^ Proportions of patients with an mRS score of three or more came from the STOP-Stroke study^22^ that identified 8.7% of LAO stroke patients with a pre-stroke mRS of 3 or more, which is consistent with reports from the study logs of trials included in HERMES.^12^ However, we update our estimates of those excluded by pre-stroke dependence to include some patients with an mRS of 3 because SSNAP data indicate EVT being performed on this subgroup. We base the percentage on the number of patients in the SSNAP national dataset treated with IVT who had a pre-stroke mRS of 3 with moderate or severe strokes (NIHSS ≥6). These patients make up 0.3% of all stroke patients, or 267 patients, which represents 3% of patients at node G (early presenters).^24^ Evidence from DEFUSE-3^10^ and the HERMES collaboration demonstrated the benefit of EVT in patients with a CT ASPECTS score of five or more. Accordingly, we reduced our estimates of patients excluded at node G by 5% (3% plus 5% less 3% to allow for a known overlap between ASPECTS and pre-stroke mRS) from 34% to 24%, increasing the proportion eligible in this group. Amongst later presenters (4.5 h–12 h), it is likely that a small number of patients with a pre-stroke mRS of 3 with an ASPECTS of 5 or better would be considered eligible. We therefore reduced, by consensus, the clinical and imaging exclusions from 75% to 72.5%. This results in an extra 210 late presenting patients progressing (to advanced imaging) from node K.

Amongst the group of EVT eligible early presenting patients, we had previously estimated a 5% spontaneous recanalisation rate (node J). Recent evidence suggests that this rate was an underestimate, and that 8.75% is a more accurate estimate.^25^ This reduces the eligible population in the early presenting group, whether advanced imaging is utilised. In the late presenting group where IVT will not be administered, the 2% spontaneous recanalisation rate observed in DAWN/DEFUSE-3 trials.

The final piece of new evidence incorporated into our decision tree concerned patients presenting between 12 and 24 h and whose eligibility must be determined by advanced imaging. Evidence from DAWN would mean that an additional 5% of patients with a moderate to severe stroke would be eligible for EVT before any spontaneous recanalisation was accounted for in this late presenting group.^9^ Amongst DAWN participants, 11.5% of patients had a moderate to severe stroke and overall, 5% were found eligible for EVT after advanced imaging.^9^ No evidence about those presenting between 12 and 16 h was incorporated into our tree from DEFUSE-3 because no data on that specific time period were reported and the broader eligibility criteria of ESCAPE^26^ were already incorporated in our decision tree for the group presenting at 6–12 h.

Given the complex nature of acute stroke services, to effectively plan for services estimates of future eligibility for EVT are required. Like the rest of Europeans, people in the UK are living longer, and this trend will continue over the next two decades. The Office of National statistics estimate that there will be 9.9 million 75 year olds in 2039, an increase from 5.8 million in 2019.^27^ Estimates of future UK stroke incidence were reported by King et al.^28^ who used a Delphi-style approach, following a review of the literature, involving experts who were aware of evidence and trends in stroke epidemiology. The most frequently chosen estimates of increased incidence for each age group were used along side the stated current incidence for each group. These estimates assume no change in the implementation and effectiveness of the stroke prevention management of risk factors such as atrial fibrillation and hypertension.

### Re-estimated annual stroke patients eligible for EVT

The updated decision tree is presented in Figure 1. The updated estimate of annual UK stroke admissions eligible for EVT is 9580–10,020 The range is described by the use, or not, of advanced imaging in early presenters: 8690 early presenters (with no use of advanced imaging) plus 1330 late presenters totalling 10,020 or 8250 early presenters (with 100% use of advanced imaging) plus 1330 late presenters totalling 9580. This is overall a minimal reduction compared with our previous estimates in 2017 of between 10,440 (advanced imaging used in all early presenters) and 10,920 (no advanced imaging in early presenters).

### Impact of demographic trends on estimates of eligibility and impact of uncertainty around key parameters

**Figure 2.**
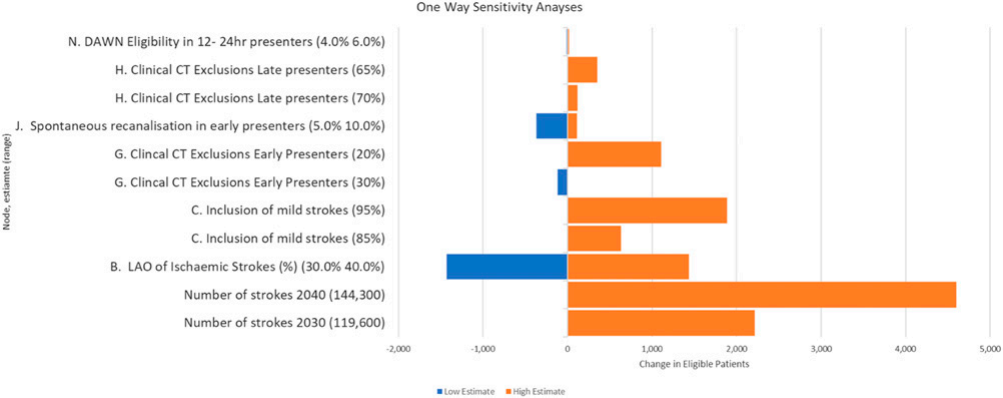
Tornado diagram of one-way sensitivity analyses for key decision nodes.

The tornado diagram (Figure 2) revealed the key nodes in the decision tree that are expected to have the biggest impact on plans for services. They were estimated assuming that advanced imaging is not used in early presenting patients. Node B, the proportion of LAOs in the stroke population, either increases or decreases the annual eligible population by 1400 patients if the proportion is increased to 40% or decreased to 30% from its assumed value of 35%. If EVT is found effective in milder stroke (NIHSS ≤5), this would increase the numbers eligible. Without an estimate of NIHSS severity of when EVT would be considered effective, we considered two scenarios: The first when 85% (an additional 5%) of early presenting LAO patients would be treated with an NIHSS of 5 or lower and the second when 95% (an additional 10%) of early presenting LAO patients would be treated with an NIHSS or 5 or lower. In these circumstances, the eligible population increased by 670 and 1980. For later presenting patients, similar reduced exclusions by 5% and 10%–70% and 65% due to NHISS score (compared to the originally estimated 75%) would increase numbers by 230 and 460 patients, respectively. Furthermore, amongst the group of early presenting patients whose NIHSS is 5 or lower are those who deteriorate within 24 h. Coutts et al.^21^ found that progression or recurrence occurred in 7% of patients presenting with NIHSS of less than 4 or a transient ischaemic attack. Our numbers could therefore be inflated to a small extent if it were found that reperfusion is indicated for these patients.

Similarly, if EVT was shown to be effective in patients with ASPECT scores of 4 or less, or in patients with a pre-stroke disability (mRs >2), the eligible population would also increase. At node G, if the exclusion rate dropped to 25% (from 29%) an additional 490 early presenting patients would become eligible. If the exclusion rate dropped to 15%, a further 1700 early presenting patients would become eligible. For later presenters, effectiveness of treatment with an ASPECT score of 4 or less would increase the eligible population by 119 if exclusion at node K dropped from 75% to 70%. If exclusion at node K dropped to 65%, then a further 350 late presenting patients would become eligible.

By 2030 and 2040, these EVT eligibility estimates could increase considerably as the UK population ages. These analyses (see Table 1) indicate that by 2030, there would be an additional 24,000 patients entering our tree and an additional 49,000 by 2040. Before any account is taken of evidence of EVT effectiveness in new sub-populations of LAO stroke, this represents approximately an additional 2400 to 4900 LAO patients per annum eligible for thrombectomy.

**Table 1.** Future Estimates of population and stroke incidence.

Age band	Population increase in 2030 from 2020	Population increase in 2040 from 2020	Mean incidence per 100,000	Increased incidence in 2030 (%)	Increased incidence in 2040 (%)	Additional stroke numbers in 2030	Additional stroke numbers in 2040
40–64	342,712	336,897	131	0	0	449	441
65–74	958,520	1,316,576	509	0	0	4879	6701
75–84	1,096,676	2,044,190	947	0	0	10,386	18,982
85–100	442,085	1,124,513	1823	5	10.5	8,462	22,652
Total	2,839,993	4,822,176	—	—	—	24,176	48,776

## Discussion

Our updated analysis of the impact of new evidence between 2018 and 2021 on the numbers of patients in the UK eligible for EVT has shown that the addition of evidence from DAWN and DEFUSE-3 resulted in a negligible impact on previous estimates of UK patients who are eligible for EVT. Both trials increased the estimates of numbers of patients eligible in absolute terms only very modestly mainly because of their strict eligibility criteria and because we had previously allowed for EVT eligibility up to 12 h after onset (whether precise onset time known or not). The shift of ASPECTS exclusion from <6 to <5 increased numbers eligible by a more substantial 5%. The inclusion of early presenting patients with moderate or severe strokes and a pre-stroke mRS of 3 or more increased annual eligibility as 3% fewer patients were deemed ineligible by this criterion. However, both increases were more than offset by a modest reduction in the assumed incidence of LAO (from 40 to 35%) due to new evidence^29^ and a revision upwards in the proportion of early presenters with recanalisation before EVT could be performed (from 5 to 8.75%). In the UK, imaging practices may affect the observed LAO rate and the previous UK estimate of 39% may reflect a smaller denominator of patients.^7^

TESLA,^14^ TENSION,^15^ MOSTE,^18^ SELECT 2,^16^ LASTE^17^ and ENDOLOW^30^ are amongst the ongoing trials whose results could appreciably alter our estimates form eligibility. These trials have been taking place during the global COVID-19 pandemic and completion and reporting of their results are likely to be delayed, potentially by up to 2 years, an additional justification to updating our 2017 eligibility estimates. TESLA, estimated to complete at very earliest in late 2022, will consider patients with ASPECTS scores of 2–5 presenting within 24 h of onset. It therefore has the possibility to impact node G of our decision tree (clinical and CT exclusions in early presenters) and eligibility in late presenters identified by advanced Imaging (nodes H and M). Based on plausible ranges, such as the percentage of late presenters who had a pre-stroke mRS of 3 and would have favourable CTs, this indicates differences in eligibility of several hundred patients per year in the late presenting group. TENSION, now estimated to complete, at very earliest, in March 2024 includes patients with an ASPECTS score of 3–5 presenting within 11 h and includes patients with low NIHSS scores as does ENDOLOW trial. These trials have the possibility to impact our estimates at nodes C, G and H. Eligibility for EVT in patients with mild strokes (NIHSS <6) has the potential to considerably increase the eligible population by 5820 at node D. Assuming that this group of patients were equally likely to present early (78%) and because lower NIHSS scores are likely to be associated with higher ASPECTS scores and mRS <3, the proportion of early presenters excluded at node G would decrease appreciably. However, it is probable that most of the low NIHSS early presenters would be lower (SSNAP reports 50% patients with mild strokes presenting within 270 min; therefore, of 5,820, 2910 would still be eligible at point J.). Recent evidence also suggests benefits in patients with a pre-existing mRS of 3 (and above) further reducing clinical exclusions. Larsson and colleagues observed that 20% of patients with pre-stroke disability receiving EVT returned to their pre-stroke functional level.^31^

SELECT 2, estimated to report at very earliest in early 2022, includes patients with an ASPECTS score of 3 or above within 24 h of ‘last known well’ and therefore has a similar potential to impact our estimates as TESLA (nodes G, H and M). LASTE, reporting at very earliest February 2022, includes early presenters with an ASPECTS score of 0–5 or 4–5 if aged 80 or over. The impact of LASTE is therefore at node G and node H.

Perhaps the most significant factor to service planners though is the effect of an ageing population, which suggests numbers of stroke patients eligible for EVT increasing by >20% in 2030 and >40% in 2040. Ageing may be associated with poorer pre-stroke health, but as there have been few participants aged 85+ in stroke EVT trials, the precise incidence of treatable LAO is unclear in this population. We also know that ageing populations are increasing more in rural areas (generally living further away from urban EVT centres), resulting in later presentation on average.

Our estimates take no account of cost effectiveness, which may influence decisions about eligibility for some groups with pre-existing disability or where EVT may reduce mortality in patients who survive with significant disability.

## Conclusion

The addition of new trial evidence from 2017 to 2021 has not substantially altered our previous estimate of stroke patients in the UK that are eligible for EVT each year. Although ongoing trials have the prospect of further considerably revising estimates of overall eligibility (mostly increasing them), the COVID-19 pandemic has delayed most of these trials appreciably and so our updated estimates and associated estimates of uncertainty are therefore an important source of information for those managing or commissioning EVT services in the next 2–3 years. Over a longer time horizon, population demographic trends appear to have the greatest impact on the incidence of LAO stroke and consequent demand for EVT services.
